# Exposure to Violence and Mental Health Outcomes Among Pre-schoolers in a South African Birth Cohort

**DOI:** 10.1007/s10802-024-01211-y

**Published:** 2024-06-11

**Authors:** Lucinda Tsunga, Jon Heron, Marilyn T. Lake, Sarah L. Halligan, Susan Malcolm-Smith, Nadia Hoffman, Heather J. Zar, Abigail Fraser, Dan J. Stein, Kirsten A. Donald

**Affiliations:** 1 Department of Paediatrics and Child Health, University of Cape Town, Cape Town, South Africa; 2 Department of Population Health Sciences, University of Bristol, Bristol, United Kingdom; 3 Department of Psychology, University of Bath, Bath, United Kingdom; 4 Department of Psychiatry, University of Stellenbosch, Stellenbosch, South Africa; 5 ACSENT Laboratory, Department of Psychology, University of Cape Town, Cape Town, South Africa; 6 Department of Psychiatry, University of Cape Town, Cape Town, South Africa; 7 The SA-MRC Unit on Child and Adolescent Health, University of Cape Town, Cape Town, South Africa; 8 Neuroscience Institute, University of Cape Town, Cape Town, South Africa; 9 South African Medical Research Council Unit on Risk & Resilience in Mental Disorders, Stellenbosch, South Africa

**Keywords:** Community violence, Domestic violence, Maltreatment, Polyvictimisation, Mental health, Pre-schoolers

## Abstract

Little is known about the relationship between violence exposure and mental health in preschoolers living in low- and middle-income countries (LMICs). Multiple regression analyses investigated associations between violence exposure and mental health in the Drakenstein Child Health Study (*N* = 978), a South African birth cohort. Lifetime violence exposure was assessed at age 4.5 years using the parent-report Child Exposure to Community Violence Checklist (CECV). Mental health was assessed at age 5 years using the Child Behaviour Checklist (CBCL 1.5–5). Eighty-three percent of the children were exposed to some form of violence. Internalising and externalising behaviours were positively associated with overall violence exposure (β per one unit change in the overall score = 0.55 [0.16, 0.94] and β = 0.53 [0.23, 0.84], respectively), domestic victimisation (β per one unit change in the subscore = 1.28 [0.28, 2.27]; β = 1.14 [0.37, 1.90]) and witnessing community violence (β = 0.77 [0.15, 1.39]; β = 0.68 [0.19, 1.18]). There was a positive association between polyvictimisation and externalising (β = 1.02 [0.30, 1.73]) but not internalising (β = 0.87 [-0.06, 1.80]) behaviour problems. Evidence for an association of witnessing domestic violence with internalising (β = 0.63 [-0.97, 2.24]) or externalising (β = 1.23 [-0.04, 2.50]) behaviours was less robust. There was no association between community victimisation and internalising or externalising behaviours (β = 0.72 [-1.52, 2.97; β = 0.68 [ -1.06, 2.41]). Observations highlight the risk for mental health problems among preschoolers living in high-violence contexts and emphasize the need for early interventions.

**Supplementary Information:**

The online version contains supplementary material available at 10.1007/s10802-024-01211-y.

## Background

Exposure to violence is one of the leading public health issues of our time, contributing to mortality, disability and poor mental health (Krug et al., 2002). When occurring in childhood, violence exposure can potentially alter developmental trajectories. Understanding how violence can impact mental health during early development may guide strategies for prevention and relevant policies that have the potential for a lasting impact on individual and societal well-being (Irwin et al., 2007). The World Health Organisation (WHO) describes two main categories of violence: (1) family and intimate partner violence (IPV) is mostly between members of the same household and intimate partners, and typically occurs in the home; (2) community violence involves unrelated individuals and generally occurs outside the home. These forms of violence can manifest as physical, sexual, or psychological abuse (Krug et al., 2002).

Exposure to violence has been found to be associated with poor mental health among school-aged children, adolescents and adults living in high-income countries (HICs). A large body of research has linked domestic victimisation to both internalising (e.g., depression, anxiety) and externalising (e.g., aggressive behaviours) behaviour problems (Baldwin et al., 2023; Carr et al., 2020; Vibhakar et al., 2019). Childhood exposure to intimate partner violence (IPV) has been linked to behavioural problems (see reviews: Fong et al., 2019; Lourenco et al., 2013) in children and adolescents. A meta-analysis investigating the relationship between community violence and mental health across individuals aged 3–25 years, found a stronger association between community violence and externalising behaviour problems than internalising behaviour problems (Fowler et al., 2009).

Studies investigating the relationship between exposure to domestic victimisation, IPV or community violence and both internalising and externalising behaviours in children, have however neglected pre-schoolers, especially those living in low- and middle-income countries (LMICs). For example, of the 114 studies reviewed by Fowler et al. (2009), only six studies assessed mental health outcomes in pre-schoolers, and only one of these studies was conducted in a LMIC setting. This omission is important, as studies investigating time-dependent effects of violence exposure across the lifespan have highlighted the preschool years as a sensitive developmental period where domestic victimisation predicts later mental health problems (Dunn et al., 2013; Kaplow & Widom, 2007; Manly et al., 2001). Importantly, children in LMICs may experience a significantly higher burden of violence than those living in HICs (Mercy et al., 2008, 2017).

Existing studies have demonstrated that polyvictimisation, i.e. exposure to multiple types of violence, further increases the risk for subsequent psychopathology (Haahr-Pedersen et al., 2020). Furthermore, a dose-response relationship has been found between the number of forms of violent experiences (i.e., domestic victimisation, IPV, peer victimisation) and psychopathology indicators, including overall psychological distress, externalising, and internalising behaviour problems (Haahr-Pedersen et al., 2020; Le et al., 2018). However, again, the evidence mainly focuses on older children and adolescents from HICs. Out of the 22 studies reviewed by Haahr-Pedersen et al. (2020) eight included pre-schoolers and only one was conducted in an LMIC. In a review of studies investigating polyvictimisation in children and adolescents in low- and lower-middle-income countries, only three studies were conducted with a preschool sample (Oh et al., 2018).

In our study, we therefore examined the relationship between lifetime exposure to violence in early childhood (assessed at 4.5 years of age) and mental health at 5 years of age in participants of the Drakenstein Child Health Study (DCHS), a longitudinal birth cohort in the Western Cape Province of South Africa. South African children experience a high burden of violence, for example, child homicide rates are more than twice the global average (Mathews et al., 2013).

First, we examined the relationship between lifetime general violence exposure and exposure to specific forms of violence assessed at 4.5 years of age with internalising and externalising behaviour problems at 5 years of age. Based on observations from older children and adolescents, we hypothesised that there would be an association between all forms of violence exposure by 4.5 years and both internalising and externalising behaviour problems at age 5. We also hypothesised that the relationship between exposure to witnessing community violence and mental health would be stronger for externalising than internalising behaviour problems based on previous literature. Second, we investigated whether there were linear dose-response relationships between polyvictimisation (i.e., the number of types of violence experienced) and internalising and externalising behaviour problems in the preschool sample.

## Method

### Study Design

The DCHS is a prospective birth cohort, that applies an interdisciplinary approach to understanding factors that influence child health and development in the Drakenstein sub-district of the Cape Winelands, Western Cape, South Africa (Zar et al., 2015). Pregnant women were recruited between 2012 and 2015 at 20–28 weeks’ gestation and mother-child dyads were prospectively followed up until at least the child was 10 years old. Here, we focussed on children’s lifetime exposure to violence assessed at age 4.5 years and internalising and externalising behaviour problems at 5 years of age.

### Study Setting

The DCHS cohort communities are characterised by relatively high levels of psychosocial risk factors, including poverty, mental health conditions, unemployment, IPV and substance use (Groves et al., 2015; Stein et al., 2015). The population is relatively stable with little migration and over 90% use the public health care systems (Zar et al., 2015), which makes these communities generally representative of other peri-urban communities in South Africa and LMICs.

### Participants

**Fig. 1:**
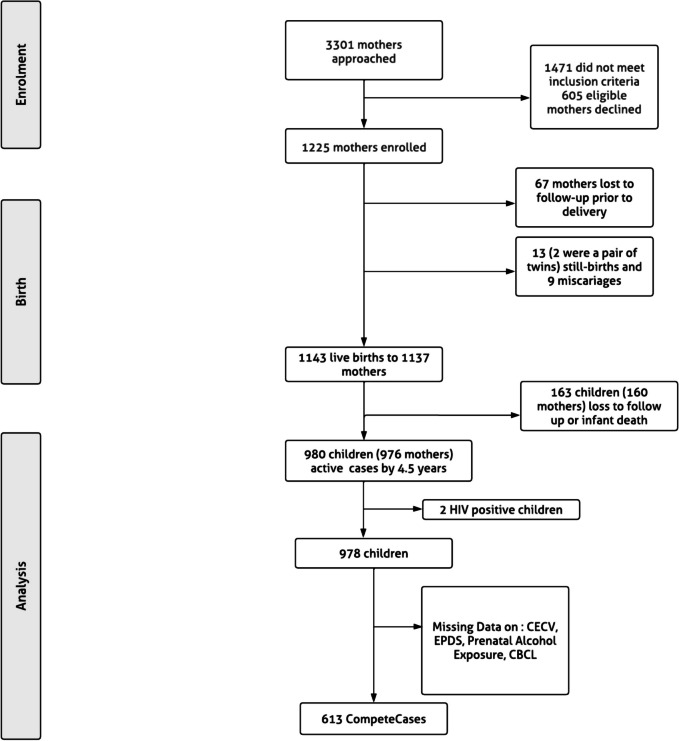
Flow chart of DCHS participation and missing data

Eligible pregnant women were at least 18 years old and attending antenatal visits at either of the two public sector primary health care clinics in the study catchment area. At enrolment, pregnant women provided informed written consent and were further re-consented annually after childbirth. Mother-child dyads attended follow-up visits at the two clinics and Paarl Hospital (Zar et al., 2015). A total of 1137 mothers and 1143 children were enrolled into the study. Currently, 980 children and 970 mothers are active in the cohort (see Fig. 1).

### Procedure

All the DCHS methods and procedures were in accordance with the Declaration of World Medical Association (2013). The Faculty of Health Sciences Research Ethics Committee, University of Cape Town (401/2009) and Stellenbosch University (N12/02/ 0002) approved the study’s protocol together with the Western Cape Provincial Research Committee (2011RP45). Trained study staff from the community guided the informed consent process and completed assessments with the mothers, in the mothers’ preferred language, isiXhosa, Afrikaans or English. Study visits were conducted in private rooms at the primary clinics, community centres or the main Hospital by trained research assistants. Where mental health conditions of safety problems were found, the study actively referred mothers and children to support services.

### Measures

DCHS maternal and child measures have been previously described (Donald et al., 2018; Stein et al., 2015) and included the following:

### Child Exposure to Violence

Violence exposure was assessed using the Child Exposure to Community Violence Checklist (CECV) which captures lifetime exposure to community and domestic violence (Amaya-Jackson, 1998). The CECV version used in this study has been adapted to fit a South African context and has shown good reliability in previous studies (Fincham et al., 2009; Kaminer et al., 2013b). The CECV was translated from English to Afrikaans and isiXhosa using a forwards and backwards approach. The translations were cross-checked by DCHS staff fluent in the relevant languages, based in the study communities, to ensure that a suitable dialect was used. Furthermore, the translation team, consisting of at least 3 mother-tongue speakers for each language, discussed translations during a meeting in order to reach consensus. Trained research assistants administered the questionnaires to caregivers in either Afrikaans or isiXhosa, and trained interpreters were used for isiXhosa-speaking respondents. Interpreters were native isiXhosa speakers and fluent English speakers. Caregivers were asked to report on 35 items assessing their child’s lifetime exposure to violence when their child was approximately 4.5 years old. The CECV response items have four levels that rate exposure frequency between “0” (never) and “3” (many times).

For our analyses we simplified the responses by combining responses above “0” into a “yes” category, indicating violence exposure, while keeping the “0” rating as an indicator of no exposure. This was done to enable us to create exposure variables that clearly reflect the number of types of violence exposures, as opposed to conflating exposure frequency and type. We used the CECV Total (sum of all 35 items, range = 0–35, Macdonald’s Omega [*ω*] = 0.91) reflecting overall violence exposure as well as four additional subscales that were previously created to capture community or domestic violence that is either witnessed by the child or directed at the child (Tsunga et al., 2023), namely, *Witnessing Community Violence* (10 items, *ω* = 0.77), *Community Victimisation* (8 items, *ω* = 0.90), *Witnessing Domestic Violence* (6 items, *ω* = 0.85) and *Domestic Victimisation* (11 items, *ω* = 0.76). Finally, we created a continuous polyvictimisation variable that captures whether a child experienced between 0 and 4 distinct types of violence, consistent with previous studies (Finkelhor et al., 2007; Kaminer et al., 2013b; Turner et al., 2010). We used continuous scores for analyses and categorical exposure status for descriptive purposes. The latter allowed us to capture prevalence patterns across varying numbers of violence types.

#### Child Mental Health

Mental health was assessed when the child was approximately 5 years old using the parent-report version of the Child Behaviour Checklist for children aged 1.5–5 years (CBCL 1.5–5; Achenbach, 2009). The CBCL consists of ninety-nine questions asking about child emotional and behavioural problems in the past 6 months on a 3-point Likert scale (0 = not true; 1 = somewhat or sometimes true; 2 = very true or often true). We used two subscales representing second-order categories of syndromes: internalising behaviour problems (36 items measuring withdrawn behaviour, somatic complaints and symptoms of anxiety/depression, score range = 0–72, *ω* = 0.92) and externalising behaviour problems (24 items measuring rule-breaking behaviour and aggressive behaviour, score range = 0–48, *ω* = 0.93). Raw scores were converted into *t-*scores with a mean of 50 and a standard deviation of 10. We used continuous *t*-scores for each subscale as is standard practice, and computed clinical classifications where non-clinical symptoms are indicated by a *t*-score ≤ 59, subclinical symptoms are indicated by *t*-scores between 60 and 64 and clinical symptoms are indicated by a *t*-score ≥ 65 (Achenbach & Rescorla, 2000). We used continuous scores for analyses and clinical categories for descriptive purposes.

#### Sociodemographic Characteristics

A questionnaire adapted from the South African Stress and Health (SASH) Study was used to gather data on household income, maternal educational background, employment status, and marital status (Myer et al., 2008). Questionnaires were administered antenatally at 28 to 32 weeks’ gestation and during annual study visits. Child biological sex was established at birth.

### Antenatal Maternal Depression

Antenatal maternal depression was assessed at 28–32 weeks’ gestation using the Edinburgh Postnatal Depression Scale (EPDS), comprised of 10 items assessing recent symptoms of depression (Cox et al., 1987). Responses were scored on a 4-point Likert scale ranging from 0 (*never*) to 3 (*yes, most of the time*), where the highest score possible was 30. We used continuous scores for analyses and clinical thresholds for descriptive purposes, classified as *Above Clinical Threshold* for scores ≥ 13 and *Below Clinical Threshold* for scores <13.

### Prenatal Substance Use

The Alcohol, Smoking and Substance Involvement Screening Test (ASSIST) was used to measure prenatal substance use (Ali et al., 2002). Alcohol use and smoking were captured by seven items indexing: lifetime use (1 item: yes/no); frequency of alcohol/smoking use in the past 3 months (3 items: using a 5-point Likert scale where responses range from rated from 0 (“never)” to 4 (“daily”); family or friends’ concern about use (1 item) and attempts to quit (1 item), responses for both items were on a 3-point Likert scale ranging from 0 (“never)” to 2 (“yes in past 3 months”). The ASSIST was administered at 28 to 32 weeks of gestation to capture alcohol and tobacco use during the past 3 months (week 14 to week 18 of pregnancy).

Mothers were further asked about their prenatal alcohol use during postpartum assessments at 3–6 weeks and 2 years. We combined the information from these three sources to create a composite indicator of prenatal alcohol use. For our analysis, prenatal exposure to alcohol or smoking is characterised by ‘exposure’ vs. ‘no exposure’. ‘Exposure’ refers to any reported alcohol or tobacco use during pregnancy, while ‘no exposure’ indicates no reported alcohol or tobacco use during pregnancy.

### HIV Exposure

Maternal HIV status was assessed prenatally at enrolment through self-report and by routine HIV testing for prevention of mother-to-child transmission, with retesting of those mothers who tested negative every 12 weeks during pregnancy, following local guidelines. HIV-exposed children (based on maternal HIV status) were repeatedly tested for HIV at 6 weeks, 9 months and 18 months, or following cessation of breastfeeding when it occurred past 18 months. Two HIV-positive children were excluded from the sample. For the HIV-negative children, we used a dichotomous child HIV exposure variable that captures whether a child was exposed to HIV or not (HIV unexposed vs. HIV exposed uninfected).

### Statistical Analysis

Descriptive statistics were used to describe participants’ demographic characteristics as well as violence exposure and mental health outcomes. Multivariable linear regression models were used to examine the relationship between lifetime violence exposure assessed at age 4.5 and mental health at 5 years, adjusting for, household income, maternal education, maternal employment status, maternal marital status, prenatal smoking exposure, prenatal alcohol exposure, maternal antenatal depression and child HIV exposure. This is because existing literature has highlighted the role that prenatal, environmental, child and maternal factors play in increasing the risk for or providing protection against mental health problems during development, with previous work having focused on HIV exposure (Wedderburn et al., 2019), alcohol (Myers et al., 2017) and tobacco exposure (Malcolm-Smith et al., 2022), and maternal depression during pregnancy (Malcolm-Smith et al., 2022; Shuffrey et al., 2022), and prenatal family socioeconomic indicators (Malcolm-Smith et al., 2022; Walker et al., 2011). As we found no strong evidence for sex interactions (only one association had *p* < 0.05 out of 12 sex interaction analyses), analyses were not stratified by sex. All analyses were conducted in R 0.2.3 GUI 1.79 High Sierra build (8198) and RStudio Version 2023.03.0+386 (2023.03.0+386).

### Missing Data

Of the 16 (confounder, exposure and outcome) variables used in the analyses, half had some degree of missing data, resulting in 613 cases with complete data from 978 dyads (see the study flow chart presented in Fig. 1 and Supplementary Fig. 1).

We conducted multiple imputation via fully conditional specification to address missing data on the CECV, CBCL, ASSIST and EPDS using the “mice” R package (van Buuren & Groothuis-Oudshoorn, 2011). Missing values on the CECV and CBCL were imputed at subscale level. A total of 25 variables were used in the imputation model, including six auxiliary variables; four CECV subscales capturing lifetime violence exposure at 3.5 years of age and two CBCL subscales capturing internalising and externalising behaviour problems at 4 years of age. All variables with missing data were imputed. We ran 100 imputed datasets. The resulting analysis sample consisted of 978 participants. Data was only imputed for participants who remain actively engaged in the study beyond the baseline assessment (i.e not lost to follow up/inactive). Model parameter estimates were an average of the results across the 100 imputed datasets, following Rubin’s rules for Multiple imputation (Rubin, 1987).

## Results

### Descriptive Statistics

Table 1 shows sample characteristics for the 978 participants in the current study. At the antenatal visit, 88% of the children came from households whose monthly income was below or equal to R5000 (258.66 USD). Only 6% of the mothers had tertiary education, 26% were currently employed and 60% were single. 30% of the children were exposed to tobacco, and 13% were exposed to alcohol prenatally, through maternal use. Approximately 22% of the children were HIV exposed but uninfected. Approximately 24% of the mothers had depressive symptoms above the clinical threshold during pregnancy. The sociodemographic characteristics are similar to those of inactive participants (see Supplementary Table 1). By the age of 4.5 years, 83% of the children were exposed to some form of violence. The most common form of violence was *Witnessing Community Violence* (74%), and the least common violence type was *Community Victimisation* (13%). With regards to exposure to domestic violence, *Domestic Victimisation* (31%) and *Witnessing Domestic Violence* (32%) each affected around one-third of children (see Supplementary Table 2 for subscale items). Approximately 26% of the children were exposed to two forms of violence, 15% were exposed to three forms of violence and 4% were exposed to all four violence subtypes. Approximately 10% of the children had internalising behaviour problems in the clinical range and approximately 4% had externalising behaviour problems in the clinical range. A similar pattern of results was observed across imputed and observed datasets (Supplementary Table 3).

**Table 1:** Sociodemographics, violence exposure and mental health descriptive statistics in imputed data (*N* = 978)

Variable	Distribution	
		Proportion/ Mean (SD)
**Child Sex**	Female	49.1%
**Household Monthly Income**	< R1000 (52.56 USD)	35.2%
	R1000-5000 (52.56 - 258.66 USD)	52.5%
	>R5000 (258.66 USD)	12.3%
**Education**	Primary	7.3%
	Some Secondary	55.4%
	Completed Secondary	31.7%
	Any Tertiary	5.6%
**Employment**	Currently Unemployed	73.8%
	Currently Employed	26.2%
**Marital status**	Single	60.4%
	Married/Cohabiting	39.6%
**Prenatal Tobacco Exposure**	Unexposed	70.4%
	Exposed	29.6%
**Prenatal Alcohol Exposure**	Unexposed	87.0%
	Exposed	13.0%
**Child HIV Exposure**	HIV Unexposed	78.0%
	HIV Exposed Uninfected	22.0%
**Maternal Antenatal Depression**	Above Clinical Threshold	23.6%
	Below Clinical Threshold	76.4%
**Any Violence**	Exposed	82.5%
**Witnessing Community Violence**	Exposed	73.8%
**Community Victimisation**	Exposed	13.1%
**Witnessing Domestic Violence**	Exposed	32.3%
**Domestic Victimisation**	Exposed	31.0%
**Polyvictimisation**	None	17.5%
	1 type	37.7%
	2 types	25.8%
	3 types	15.0%
	4 types	4.0%
**Internalising Behaviour Problems**	Mean (SD)	43.7 (13.0)
	Subclinical problems	5.6%
	Clinical symptoms	10.3%
	Non-clinical symptoms	84.1%
**Externalising Behaviour Problems**	Mean (SD)	41.3 (10.4)
	Subclinical symptoms	2.9%
	Clinical symptoms	3.6%
	Non-clinical symptoms	93.5%

### Violence Exposure and Mental Health

Table 2 presents unadjusted and adjusted multivariable linear regression results for the associations between violence exposure and mental health. In adjusted models, there was an association between *Overall Violence Exposure, Witnessing Community Violence* and *Domestic Victimisation* and both internalising and externalising behaviour problems in children. By contrast, associations of *Witnessing Domestic Violence* or *Community Victimisation* with internalising or externalising behaviour did not reach statistical significance at this age in this cohort. Every additional type of violence to which children were exposed was associated with a 0.87-point higher score on internalising behaviour problems, though the confidence interval spanned the null value (95% CI = -0.06, 1.80). There was an association between polyvictimisation and externalising behaviour, where each additional type of violence experienced corresponded to a 1.02-point increase in externalising behaviour problem score.

**Table 2:** Results of adjusted linear regression analyses examining violence exposure and internalising and externalising behaviour problems in imputed data

*N* = 978		**Internalising Behaviour Problems**				**Externalising Behaviour Problems**			
		β	SE	95% CI	*p-*value	β	SE	95% CI	*p-*value
**Overall Violence Exposure**	Unadjusted	0.70	0.18	0.33, 1.06	< 0.001	0.77	0.15	0.48, 1.06	< 0.001
	Adjusted*	0.55	0.20	0.16, 0.94	0.005	0.53	0.15	0.23, 0.84	0.001
**Witnessing Community Violence**	Unadjusted	0.94	0.29	0.37, 1.51	0.001	1.04	0.24	0.57, 1.50	< 0.001
	Adjusted	0.77	0.32	0.15, 1.39	0.016	0.68	0.25	0.19, 1.18	0.007
**Community Victimisation**	Unadjusted	1.12	1.14	-1.13, 3.35	0.326	0.96	0.90	-0.81, 2.72	0.286
	Adjusted	0.72	1.14	-1.52, 2.97	0.527	0.68	0.88	-1.06, 2.41	0.442
**Witnessing Domestic Violence**	Unadjusted	1.09	0.80	-0.48, 2.66	0.175	1.89	0.64	0.62, 3.16	0.004
	Adjusted	0.63	0.82	-0.97, 2.24	0.439	1.23	0.65	-0.04, 2.50	0.058
**Domestic Victimisation**	Unadjusted	1.67	0.49	0.70, 2.64	< 0.001	1.62	0.39	0.86, 2.38	< 0.001
	Adjusted	1.28	0.51	0.28, 2.27	0.012	1.14	0.39	0.37, 1.90	0.004
**Polyvictimisation**	Unadjusted	1.30	0.45	0.42, 2.18	0.004	1.60	0.35	0.91, 2.28	< 0.001
	Adjusted	0.87	0.47	-0.06, 1.80	0.066	1.02	0.37	0.30, 1.73	0.005

All adjusted models were adjusted for sex, household monthly income, maternal education, maternal employment status, maternal marital status, prenatal smoking exposure, prenatal alcohol exposure, maternal antenatal depression and HIV exposure

*SE* Standard Error, *CI* Confidence Interval, *p* *p*-value

Linear regression models based on complete case analysis with a sample of 613 participants were similar to the models based on multiple imputation (estimates were of a similar magnitude, see Supplementary Table 4 for adjusted models and Supplementary Table 5 for unadjusted models in observed data).

## Discussion

We examined the relationship between lifetime exposure to violence assessed at 4.5 years and mental health outcomes evaluated at 5 years in a sample of South African preschoolers and found that overall violence exposure as well as specific forms of violence namely, *Witnessing Community Violence* and *Domestic Victimisation* were associated with both internalising and externalising behaviour problems after adjusting for confounding variables. Further, we found an association of polyvictimisation with externalising behaviours, with weaker evidence for an association with internalising behaviour problems. The association between *Witnessing Domestic Victimisation* or *Community Victimisation* and mental health problems did not reach statistical significance in our sample at this age.

A majority (83%) of children in our sample had a lifetime history of exposure to violence at the age of 4.5, with *Witnessing Community Violence* (74%) being the most prevalent form of exposure. Similarly, a previous study found that, witnessed traumatic events were one of the most common forms of trauma in a sample of South African adults (Atwoli et al., 2013). Furthermore, there was evidence of polyvictimisation in this young sample, with 45% of the children being exposed to at least two subtypes of violence by 4.5 years. Overall, the high rates of violence exposure observed in this sample are similar to those reported in studies conducted in South Africa and other LMICs with older children (Hayati Rezvan et al., 2021; Kaminer et al., 2013a; Laurenzi et al., 2020). Lower rates of *Community Victimisation* here than in other studies in South Africa(Kaminer et al., 2013b; Falconer et al., 2020) are likely due to the young age of children in our sample – at an earlier age, children are less likely to be unaccompanied in the community and so may be more protected. These findings highlight the burden of violence among South African children from representative peri-urban communities.

Our finding of an association between *Witnessing Community Violence* and both internalising and externalising behaviour problems is similar to the results reported in the meta-analysis by Fowler et al. (2009). They documented associations between community violence and both internalising and externalising behaviour problems across individuals aged 3 to 25 years, largely from HICs and skewed towards older age groups compared to preschoolers. Whereas they found that the association of exposure to community violence with externalising behaviour problems was more robust than with internalising behaviour, we report a comparable association with both outcome groups.

Similar to our finding that *Domestic Victimisation* was associated with mental health problems (both internalising and externalising behaviour problems), many studies have established childhood domestic victimisation as a risk factor for mental health problems in school-going children, adolescents and adults (Carr et al., 2020; Gershoff & Grogan-Kaylor, 2016; Li et al., 2016; Vibhakar et al., 2019). However, these studies primarily included populations living in HICs. The widespread normalisation of corporal punishment as a form of discipline in the home in South Africa is therefore of concern (Mathews et al., 2014).

Whilst evidence for the association between *Witnessing Domestic Violence* exposure and externalising behaviour problems was less robust in this cohort, prior work has found more consistent evidence for this relationship (see review: Fong et al., 2019). Given the established link between witnessing domestic violence and internalising symptoms(see review: Vu et al., 2016), the failure of this association to reach statistical significance in our sample may reflect difficulties in recognizing or reporting such behaviours, or the emergence of such behaviours later in childhood.

The relationship between polyvictimisation and child behaviour problems at age 5 years, highlights the role that exposure to multiple types of violence plays in increasing the risk of psychopathology. These findings are suggestive of the added impact of an increased burden of violence on children’s mental health, similar to the findings of Haahr-Pedersen et al. (2020). These authors described a strong association between polyvictimisation and various psychopathology indicators, including externalising and internalising behaviour problems in children aged 0–17 in their review, largely consisting of children from HIC settings. Furthermore, polyvictimisation emerged as a stronger risk factor for mental health problems than individual types of victimisation. Another review focusing on children and adolescents up to 19 years of age in LMICs, similarly found a link between polyvictimisation and an increased risk of mental health problems (Le et al., 2018). Nevertheless, their sample largely consisted of children much older than those in our sample. Polyvictimisation likely increases allostatic load, such that the body experiences cumulative physiological damage in response to multiple stressors over time. This results in dysfunction of various regulatory systems including behavioural functioning (Danese & McEwen, 2012). Furthermore, a previous study conducted in the USA identified four pathways to polyvictimisation, including the presence of emotional problems in young children (< 9 years) and living in dangerous communities, high-adversity homes, and violent and conflict-ridden families in children 10–17 years old. Furthermore, polyvictimisation onset was associated with starting elementary and high school (Finkelhor et al., 2009). These findings highlight the substantial burden of violence experienced by children in our sample in comparison, with polyvictimisation occurring by age 4.5 in the context of low socio-economic indicators and violent settings.

The rates of clinically significant psychopathology in our sample were lower than those found in a previous meta-analysis investigating the effects of family violence on children’s behaviour (Sternberg et al., 2006). They found that 28 − 50% of the children aged 4–14 years fell within the clinical range of behaviour problems. There are several possible explanations of this inconsistency. It is plausible that the low rates of clinically significant psychopathology observed in the present study are a true finding, emphasizing that children exposed to traumas may be resilient (van Breda & Theron, 2018). Whilst, similar to the present study, Sternberg et al’s. (2006) metanalysis used caregiver reports to assess children’s mental health problems, 31% of their reviewed studies used state records to assess children’s exposure to violence. This contrasts with the current study’s sole use of caregiver reports to assess children’s violence exposure. This variation in data sources potentially explains the different findings. It is also possible, however, that in our sample there are difficulties in recognizing or reporting such behavioural problems given the sample’s young age (Poulou, 2015), or that such behaviours emerge later in childhood (McCrory et al., 2017). Indeed, Sternberg et al’s. (2006) reviewed sample predominantly comprised of school-going children and adolescents.

This study has a number of strengths including the use of a prospective longitudinal birth cohort sample from South Africa, with high retention rates. This allowed us to contribute to the literature in LMICs where children experience high levels of violence but are understudied. Furthermore, our study examined the associations between violence exposure and mental health outcomes in early childhood, while the majority of the previous research focused on these associations later in life. This allowed us to identify the effects of exposure to violence in early childhood when it occurs. We also captured direct and indirect exposure to domestic and community violence, providing us with comprehensive findings. Investigating polyvictimisation allowed us to capture this prevalent phenomenon (Suliman et al., 2009; Williams et al., 2007). We also adjusted for several important potential confounding factors.

However, it is important to interpret the results of our study within the context of several limitations. Firstly, reports of children’s exposure to violence as well as their behaviour were given by the same caregiver, typically the mother. This may have led to shared rater bias, where obtaining information on exposure and outcomes from the same reporter may inflate the associations between the variables. Secondly, caregivers may have underreported children’s violence exposure and mental health problems due to social desirability bias (Lagattuta et al., 2012). The use of caregivers as reporters is common in this age group given that young children may not be able to adequately describe their traumatic experiences nor have insight into their behaviours. However, the widespread normalisation of certain forms of violence in South Africa, such as the use harsh disciplinary practices (Mathews et al., 2014), mean that social desirability bias may have impacted the reporting of some but not all types of violence measured. Thirdly, whilst there was relatively little attrition in the study overall, there was missing data on key variables at the time points studied. We addressed this through multiple imputations with an adequate number of datasets and capitalised on auxiliary variables available through the repeated measures design of the DCHS. Fourthly, although we adjusted for various potential confounders in our analyses, residual confounding cannot be excluded. Lastly, we note that given that our violence exposure subscales differ in the number of CECV items they comprise which limits their comparability to each other in their associations with mental health outcomes in terms of magnitude of effect size estimates.

Our findings emphasize the need for strategies that prioritise interventions aimed at both reducing the burden of violence on children living in these contexts as well as therapeutic interventions for those affected. This is especially important for younger children who have not yet entered school. Poor mental health may affect capacity to cope with academic demands at school (Romano et al., 2015) which in turn has been linked to poor educational trajectories and is a long-term contributor to poverty (Fry et al., 2018; Jaffee et al., 2018; Tafere, 2017).

Exposure to violence in South African communities may be rooted in socioeconomic inequalities as well as a colonial past that fostered domestic and community violence (Bruce et al., 2007; Mathews et al., 2014; Ward et al., 2013). Interventions targeted at the societal level are needed to tackle these systemic problems and stop the cycle of violence in these communities. Notably, the mothers of the children in this sample reported high rates of exposure to trauma in their own lives (Barnett et al., 2018). Furthermore, associations between domestic victimisation and poor mental health outcomes in this young sample emphasize the need for parenting interventions to eradicate the use of harsh discipline. Future research needs to also investigate protective factors in children living in contexts such as the Drakenstein with the aim of understanding how to boost resilience.

## Conclusion

We found associations between lifetime exposure to domestic victimisation, witnessing community violence, and polyvictimisation assessed at age 4.5 years and internalising and externalising behaviour problems at age 5 years in our sample. Our findings emphasise violence exposure as a human rights and public health issue in young children in LMICs. We also highlight the importance of investigating protective factors that contribute to resilience in children exposed to violence given that despite the high levels of violence children in our sample experienced, the majority of them did not exhibit clinically significant mental health problems.

## Electronic Supplementary Material

Below is the link to the electronic supplementary material.

Supplementary Material 1 (DOCX 248 KB)

## Data Availability

Data can be made available on request. Collaborations for the analysis of data are welcome; the parent study has a large and active group of investigators and postgraduate students and many have successfully partnered with students or researchers from other institutions. Researchers who are interested in collaborations can find more information on our website [http://www.paediatrics.uct.ac.za/scah/dclhs]*.*
