# Supplementary material for: Exposure to Violence and Mental Health Outcomes Among Pre-schoolers in a South African Birth Cohort
Source: Res Child Adolesc Psychopathol. 2024 Jun 11;52(10):1635–46. doi: 10.1007/s10802-024-01211-y (PMC11461604; doi:10.1007/s10802-024-01211-y)
Supplement: Supplementary file 1 — Supplementary Material 1 (DOCX 248 KB) [file 10802_2024_1211_MOESM1_ESM.docx]

Supplementary

*Supplementary Table 1 Characteristics of active and inactive samples*

|  |  | Active sample (N = 978)* | Inactive sample (N = 163) |
| --- | --- | --- | --- |
|  |  | Proportion | Proportion |
| Child Sex | Female | 49.1% | 47.2% |
| Household Monthly Income | < R1000 (52.56 USD) | 35.2% | 25.9% |
|  | R1000-5000 | 52.5% | 50.6% |
|  | >R5000 (258.66 USD) | 12.3% | 23.5% |
| Education | Primary | 7.3% | 7.5% |
|  | Some Secondary | 55.4% | 41.6% |
|  | Completed Secondary | 31.7% | 39.8% |
|  | Any tertiary | 5.6% | 11.2% |
| Employment | Unemployed | 73.8% | 68.1% |
| Marital Status | Married/cohabiting | 39.6% | 44.8% |
| Prenatal Tobacco Exposure | Exposed | 29.6% | 21.0% |
| Prenatal Alcohol Exposure | Exposed | 13.0% | 11.6% |
| Child HIV Birth Exposure | HIV exposed uninfected | 22.0% | 19.6% |
| Maternal Antenatal Depression | Above clinical threshold | 29.6% | 24.4% |

*All values were observed

*Supplementary Table 2 Violence Exposure Subscales and Corresponding CECV Items*

| Subscale | Item |
| --- | --- |
| *Witnessing Community Violence* | |
|  | Has the child ever heard gunshots? |
|  | Has the child ever seen someone beaten up in the neighbourhood? |
|  | Has the child ever seen a dead body in the neighbourhood? |
|  | Has the child ever seen somebody point a gun at another in the neighbourhood? |
|  | Has the child ever seen somebody get shot in the neighbourhood? |
|  | Has the child ever seen somebody point a knife at another in the neighbourhood? |
|  | Has the child ever seen somebody get stabbed in the neighbourhood |
|  | Has the child ever seen someone forced to do something sexual neighbourhood? |
|  | Has the child ever child known someone killed by another? |
|  | Has the child ever seen someone being killed by another person elsewhere? |
| *Community Victimisation* | |
|  | Has your house ever been robbed whilst the child was present? |
|  | Has someone ever threatened to beat up the child at school or creche? |
|  | Has someone ever threatened to beat up the child elsewhere? |
|  | Has the child been beaten up elsewhere? |
|  | Has someone elsewhere ever threatened to kill the child? |
|  | Has someone at school or creche ever threatened to shoot or stab the child? |
|  | Has someone elsewhere ever threatened to shoot or stab the child? |
|  | Has someone ever shot or stabbed the child elsewhere? |
| *Witnessing Domestic Violence* | |
|  | Has the child ever seen grownups at home hit each other? |
|  | Has the child ever seen somebody point a gun at another at home? |
|  | Has the child ever seen someone at home get stabbed? |
|  | Has the child ever seen someone at home get shot? |
|  | Has the child ever seen someone forced to do something sexual? |
|  | Has the child ever seen someone being killed by another person at home? |
| *Domestic Victimisation* | |
|  | Has someone threatened to beat up the child at home? |
|  | Has the child ever been beaten up at home? |
|  | Has someone at home ever threatened to kill the child? |
|  | Has a family member threatened to shoot or stab the child? |
|  | Has someone shot or stabbed the child at home? |
|  | Has someone made the child do something sexual? |
|  | Does someone at home shout at the child fiercely and loudly? |
|  | Has anyone at home ever used a stick or belt or hard item to hit the child? |
|  | Has anyone at home ever hit the child so hard they were hurt? |
|  | Has anyone at home ever said the child would be sent away or kicked out? |
|  | Has anyone at home ever called the child horrible names? |

*Supplementary Table 3 Descriptive statistics and comparison between observed and imputed samples*

| **Variable** | | **n (%) data missing** | **Proportion** | |
| --- | --- | --- | --- | --- |
|  |  |  | Observed data | Imputed data |
| **Prenatal alcohol** | No exposure | 41 (4.2%) | 87.1% | 87.0% |
|  | Exposure |  | 12.9% | 13.0% |
| **Maternal Antenatal Depression** | Above clinical threshold | 107 (10.9%) | 23.8% | 23.6% |
|  | Below clinical threshold |  | 76.2% | 76.4% |
| **Any Violence Exposure** | Unexposed | 233 (23.8%) | 17.6% | 17.5% |
|  | Exposed |  | 82.4% | 82.5% |
| **Witnessing Community Violence** | Unexposed | 233 (23.8%) | 25.5% | 26.2% |
|  | Exposed |  | 74.5% | 73.8% |
| **Community Victimisation** | Unexposed | 232(23.7%) | 87.3% | 86.9% |
|  | Exposed |  | 12.7% | 13.1% |
| **Witnessing Domestic Violence** | Unexposed | 232(23.7%) | 67.3% | 67.7% |
|  | Exposed |  | 32.7% | 32.3% |
| **Domestic Victimisation** | Unexposed | 232(23.7%) | 68.6% | 69.0% |
|  | Exposed |  | 31.4% | 31.0% |
| **Polyvictimisation** | 1 type | 233 (23.8%) | 37.9% | 37.7% |
|  | 2 types |  | 24.7% | 25.8% |
|  | 3 types |  | 15.4% | 15.0% |
|  | 4 types |  | 4.4% | 4.0% |
|  | None |  | 17.6% | 17.5% |
| **Internalising behaviour problems** | Borderline clinical | 127 (13.0%) | 5.6% | 5.6% |
|  | Clinical |  | 10.3% | 10.3% |
|  | Healthy |  | 84.0% | 84.1% |
| **Externalising behaviour problems** | Borderline clinical | 127 (13.0%) | 2.9% | 2.9% |
|  | Clinical |  | 3.6% | 3.7% |
|  | Healthy |  | 93.4% | 93.5% |

*Supplementary Table 4 Results of linear regression analyses examining violence exposure on internalising and externalising behaviour problems with observed data*

| *N* = 613 |  | Internalising Behaviour Problems | | | | Externalising Behaviour Problems | | | |
| --- | --- | --- | --- | --- | --- | --- | --- | --- | --- |
|  |  | β | SE | 95% CI | *p-*value | β | SE | 95% CI | *p-*value |
| Overall Violence Exposure | Unadjusted | 0.55 | 0.2 | 0.16, 0.94 | 0.005 | 0.69 | 0.16 | 0.38, 1.00 | <0.001 |
|  | Adjusted* | 0.45 | 0.21 | 0.03, 0.87 | 0.035 | 0.47 | 0.17 | 0.14, 0.79 | 0.005 |
| Witnessing Community Violence | Unadjusted | 0.60 | 0.32 | -0.03, 1.24 | 0.061 | 0.78 | 0.26 | 0.27, 1.28 | 0.003 |
|  | Adjusted | 0.51 | 0.35 | -0.18, 1.19 | 0.146 | 0.45 | 0.27 | -0.09, 0.98 | 0.101 |
| Community Victimisation | Unadjusted | 1.10 | 1.2 | -1.25, 3.46 | 0.358 | 0.88 | 0.96 | -1.01, 2.77 | 0.362 |
|  | Adjusted | 0.55 | 1.20 | -1.81, 2.92 | 0.647 | 0.44 | 0.95 | -1.42, 2.30 | 0.641 |
| Witnessing Domestic Violence | Unadjusted | 1.00 | 0.83 | -0.63, 2.62 | 0.228 | 2.02 | 0.66 | 0.72, 3.31 | 0.002 |
|  | Adjusted | 0.66 | 0.85 | -1.00, 2.32 | 0.434 | 1.39 | 0.66 | 0.08, 2.69 | 0.037 |
| Domestic Victimisation | Unadjusted | 1.60 | 0.52 | 0.58, 2.61 | 0.002 | 1.70 | 0.41 | 0.89, 2.52 | <0.001 |
|  | Adjusted | 1.26 | 0.53 | 0.22, 2.31 | 0.018 | 1.23 | 0.42 | 0.41, 2.05 | 0.003 |
| Polyvictimisation | Unadjusted | 1.00 | 0.48 | 0.06, 1.95 | 0.038 | 1.50 | 0.38 | 0.74, 2.25 | <0.001 |
|  | Adjusted | 0.64 | 0.51 | -0.36,1.64 | 0.208 | 0.93 | 0.4 | 0.15, 1.71 | 0.019 |

Note: *Each model is adjusted for sex, household monthly income, maternal education, maternal employment status, maternal marital status, prenatal smoking exposure, prenatal alcohol exposure, maternal antenatal depression and HIV exposure. *SE* = Standard Error *CI* = Confidence Interval; *N* = 613


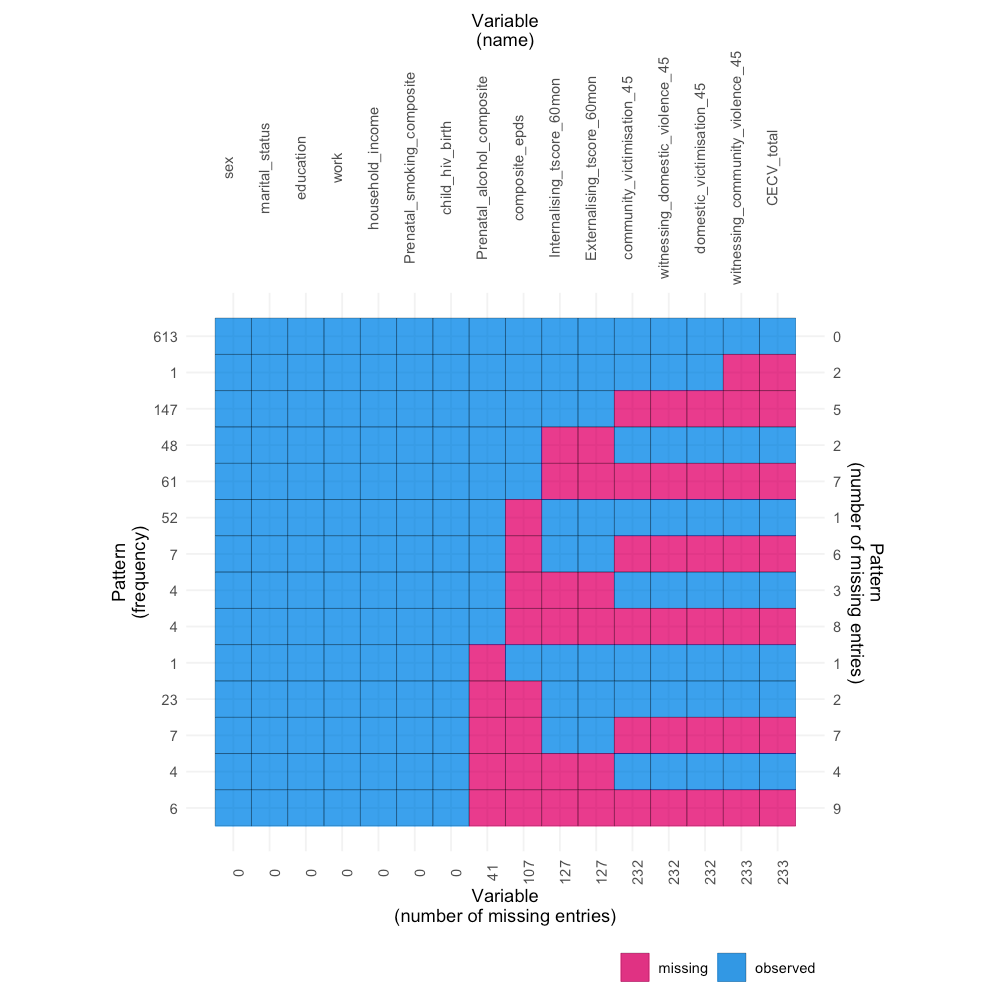


*Supplementary Figure 1 Missing data patterns: sex (Child sex), marital_status (Maternal marital status), education (Maternal Highest Level of Education), work (Maternal Employment Status), household_income (Household Income), Prenatal_smoking_composite (Prenatal Smoking Exposure), Prenatal_alcohol_composite (Prenatal Alcohol Exposure), child_hiv_birth (Child HIV Exposure), composite_epds (Maternal Antenatal Depression), Internalising_tscore_60mon (Internalising behaviour problems), Externalising_tscore_60mon (Externalising behaviour problems), community_victimisation (Community Victimisation), witnessing_domestic_violence (Witnessing Domestic Violence), domestic_victimisation (Domestic Victimisation), witnessing_community_violence (Witnessing Community Violence), CECV_total (CECV Total Score)*
